# Spatiotemporal and Behavioral Patterns of Men Who Have Sex With Men Using Geosocial Networking Apps in Shenzhen From Mobile Big Data Perspective: Longitudinal Observational Study

**DOI:** 10.2196/69569

**Published:** 2025-03-20

**Authors:** Lan Wei, Yongsheng Wu, Lin Chen, Jinquan Cheng, Jin Zhao

**Affiliations:** 1 Shenzhen Center for Disease Control and Prevention Shenzhen China

**Keywords:** men who have sex with men, geosocial networking apps, distribution, heterogeneity, mobility, HIV testing, risk behavior, big data

## Abstract

**Background:**

The use of geosocial networking apps is linked to increased risky sexual behaviors among men who have sex with men, but their relationship with HIV and other sexually transmitted infections remains inconclusive. Since 2015, the prevalence of app use among men who have sex with men in Shenzhen has surged, highlighting the need for research on their spatiotemporal and behavioral patterns to inform targeted prevention and intervention strategies.

**Objective:**

This study aims to investigate the population size, spatiotemporal and behavioral patterns, and mobility of app-using men who have sex with men in Shenzhen using mobile big data. The goal is to inform enhanced and innovative intervention strategies and guide health resource allocation.

**Methods:**

By leveraging mobile big data application technology, we collected demographic and geographic location data from 3 target apps—Blued (Blued Inc), Jack'd (Online Buddies Inc), and Zank (Zank Group)—over continuous time periods. Spatial autocorrelation (Global Moran I) and hot spot analysis (Getis-Ord Gi) were used to identify the geographic clusters. The Geodetector tool (Chinese Academy of Sciences) was adopted to measure spatially stratified heterogeneity features.

**Results:**

From September 2017 to August 2018, a total of 158,387 males aged 15-69 years in Shenzhen used one of the 3 apps, with the majority (71,318, 45.03%) aged 25-34 years. The app user-to-male ratio was approximately 2.6% among all males aged 15-69 years. The estimated population of app-using men who have sex with men in Shenzhen during this period was 268,817. The geographic distribution of app-using men who have sex with men in Shenzhen was clustered, with hot spots primarily located in central and western Shenzhen, while the distribution of HIV testing and counseling was more concentrated in central-eastern Shenzhen. Approximately 60,202 (38%) app-using men who have sex with men left Shenzhen during the Spring Festival, and 37,756 (62.7%) of them returned after the holiday. The destination distribution showed a relatively centralized flow throughout the country, with the largest mobility within Guangdong province (67.7%), followed by lower mobility to Hunan province (7.9%) and other neighboring provinces (3%-5%), such as Jiangxi, Guangxi, and Hubei Provinces.

**Conclusions:**

Shenzhen has a large population of men who have sex with men. The variation and inconsistent spatiotemporal distribution of app use and HIV testing and counseling emphasize the need to adapt traditional venue-based prevention and intervention to identified hot spots and to launch outreach initiatives that extend beyond traditional healthcare settings. Given the relatively high internal and interprovincial mobility of app-using men who have sex with men, further smartphone-based behavioral monitoring could provide valuable insights for developing enhanced and innovative HIV prevention and intervention strategies. Moreover, our study demonstrates the potential of mobile big data to address critical research gaps often overlooked by traditional methods.

## Introduction

Since the first case of AIDS was reported among men who have sex with men in the United States in 1981, AIDS has remained a major global public health issue. As of the end of 2023, it is estimated there are 39.9 million people living with HIV, with 1.3 million newly infected [1]. According to the Chinese Center for Disease Control and Prevention (CDC), there were 1.22 million people living with HIV in China by the end of 2022, with 107,000 new cases reported that year. The proportion of newly reported HIV or AIDS cases transmitted through men who have sex with men has grown rapidly in the past decades, rising from 9.1% in 2009 to 25.6% in 2022 [2,3]. Men who have sex with men have emerged as a significant high-risk group globally, with a likelihood of acquiring HIV that is 28 times greater than that of the general population [4]. In China, the HIV prevalence among this population has risen from 1.4% in 2005 to 6% in 2021 [5,6], raising concerns about the sustained effectiveness of current public health initiatives and underscoring the need for targeted interventions.

With the rapid development of the internet and social media, men who have sex with men can connect with others through various web-based platforms, including smartphone-based geosocial networking (GSN) apps. Unlike traditional fixed-location social networking, GSN apps effectively bridge online and offline dating functions, facilitating the process of seeking relationships or casual encounters. This convenience and privacy have led to the rising popularity of these apps, particularly in countries or regions with low levels of social acceptance of homosexuality. GSN apps designed for men who have sex with men, such as Grindr and Jack'd, have rapidly gained popularity in Western countries since 2009 [7]. Subsequently, several other apps including Blued and Aloha, emerged and have become increasingly popular among Chinese men who have sex with men since 2013 [8]. Blued is the first and most popular gay dating app of its kind in China. Its global users have increased from 27 million in 2015 to 49 million in 2020, with approximately 70% of users being Chinese [9].

Prior research found about 66-67% of the men who have sex with men in China used GSN apps to seek sexual partners within the past 6 months [10,11]. Our previous cross-sectional study revealed a sharp increase in app use among men who have sex with men in Shenzhen, with the rate surging from 12.5% in 2015 to 52.6% in 2017 [12]. The rapid growth of gay dating apps suggests that they have become one of the primary means for seeking sexual partners in the Chinese men who have sex with men community. Literature has documented that the use of GSN dating apps is associated with both increased risky sexual behaviors (eg, multiple sexual partners, condomless anal intercourse, drug use) and risk-reduction behaviors (eg, HIV testing and consistent condom use), although their impact on the risk of HIV and other sexually transmitted infections remains inconclusive [12-14].

Currently, the HIV testing rate in China remains below UNAID’s first 95% target, with only 78.7% of men who have sex with men having been tested for HIV, despite concerted efforts of the government, community organizations, and nongovernmental organizations [3]. Recent economic development in China has accelerated population mobility, which is recognized as an important driver for HIV transmission and a potential barrier to HIV care and treatment [15-17]. Prior research reported that the HIV epidemics in Shenzhen were predominantly driven by the migrant population [18]. Most previous studies and intervention programs relied on sentinel surveillance or venue-based populations, resulting in limited coverage of young and mobile populations. Therefore, there is a pressing need for further research into the crucial HIV-related behaviors of these marginalized groups, particularly concerning mobility patterns and access to HIV testing.

With the growing accessibility of the internet and rapid advancements in technology, mobile big data—encompassing aggregated demographics, social behavior, and geographic location information of users—has become a valuable tool for research, particularly in understanding HIV prevention, behavioral patterns, spatial dynamics, and migration trends among men who have sex with men. Earlier studies have used GSN apps to explore various aspects of the men who have sex with men including demographic characteristics, sexual practices, HIV testing, Pre-exposure prophylaxis (PrEP) use, and geospatial distribution, however, most of them were from developed countries or regions [19-23]. In China, domestic research on men who have sex with men and GSN app use primarily focused on risky sexual behavior, HIV infection, and sexually transmitted infections relying on self-reported data [24-27], which may introduce biases due to underreporting or overreporting of sensitive behaviors. Some recent studies explored spatial distribution, migration, and PrEP adherence among men who have sex with men through GSN apps [28,29]. However, most of these studies did not directly use mobile big data.

Shenzhen, designated as China’s first special economic zone in 1979, has transformed from a small village into a metropolis with a population of over 15 million. With a massive influx of internal migrant workers from across the country, Shenzhen now has the largest proportion of immigrants in China, with 66% of its population comprised of internal migrants [30]. The relatively open and inclusive cultural environment of Shenzhen attracted many men who have sex with men from across the country. Surveillance data from 2020 revealed that 62.6% of newly diagnosed HIV or AIDS cases in Shenzhen were attributed to male-to-male sexual transmission, exceeding the national average [31]. A previous cross-sectional study estimated that the active population of men who have sex with men in Shenzhen reached approximately 150,000 in 2014 [31]. Our venue-based survey showed the proportion of men who have sex with men using gay dating apps in Shenzhen has rapidly increased since 2015 [12]. The demographic context of Shenzhen, with its significant migrant population and a high proportion of men who have sex with men using gay dating apps, presents unique challenges for effective HIV prevention and interventions within this group. By leveraging large-scale mobile big data, this study aimed to investigate the actual population size, spatiotemporal and behavioral patterns, and mobility of GSN app-using men who have sex with men in Shenzhen, to gain deeper insights into this key population and inform the development of enhanced and innovative strategies for HIV prevention and interventions.

## Methods

### Setting

Mobile big data possesses characteristics such as wide coverage, real-time processing, and continuity, allowing for continuous capture of mobility information from the target population. In this study, we used a longitudinal observational design and used mobile big data app technology, specifically structured query language, to gather information on gay dating app users in Shenzhen through the platform as a service (PaaS) and further analyzed the app use patterns, the geographic distribution of app use and HIV testing and counseling (HTC) of the population over a continuous time: from September 2017 to August 2018, leveraging mobile signaling data.

In addition, as the most important traditional festival in China, the Spring Festival (Chinese New Year) is a time for family reunions, cultural celebrations, and various customs. The unique cultural significance makes it an ideal period for examining population mobility as most people travel to their hometowns. Therefore, to better understand the mobility patterns of the app-using men who have sex with men, we analyzed the geographic location changes of the population in Shenzhen before (January 1-31), during (February 1-28), and after (March 1-31) the Spring Festival, and the destination cities where these men who have sex with men traveled during the Spring Festival in 2018.

### Participants

According to guidelines for HIV prevention interventions among men who have sex with men from the China CDC, the three most frequently used apps among men who have sex with men are Blued (Blued Inc), Jack'd (Online Buddies Inc), and Zank (Zank Group) [32]. Our preliminary survey also found that approximately 97.2% of men who have sex with men in Shenzhen used Blued [12]. Therefore, all users of the target 3 apps (Blued, Jack'd, or Zank) in Shenzhen were recruited as participants, while only those males aged 15 to 69 years were included for further analysis in this study.

### Assessments

The mobile big data collected and the outcomes in this study are included as follows.

- User demographics: Information such as age, sex, and possibly other relevant characteristics of users of Blued, Jack'd, and Zank.
- Geographic location data: Information on the geographic distribution of app users, including district and subdistrict where the apps are accessed during the three defined time periods on weekdays (Monday to Friday): work time (9 AM-6 PM), social time (8 PM-2 AM), and home time (2 AM-6 AM). Additionally, data on the geographic distribution of users’ visits to free the HTC sites was collected, including users’ visits to city- or district-level (CDC) during the same 3 time periods on weekdays (Monday to Friday), and their visit to Shenzhen 258-Rainbow Working Group (258-RWG) during work time (9 AM-6 PM) on Wednesday and Sunday. 258-RWG is a nongovernmental organization composed of men who have sex with men volunteers, with technical support from Shenzhen CDC. Its initiatives such as outreach, behavioral interventions, peer education, and voluntary counseling and testing to promote prevention and mitigate high-risk behaviors. Both 258-RWG and Shenzhen CDC are the key health service providers for men who have sex with men in Shenzhen, offering free HTC, which is crucial for understanding men who have sex with men’s engagement with HIV-related services.
- Mobile signaling data: Information on the distribution of app-using men who have sex with men at the subdistrict level in Shenzhen before the Spring Festival (January 1-31, 2018), the destination cities of those who left Shenzhen during the Spring Festival (February 1- 28, 2018), and the distribution of app-using men who have sex with men after the Spring Festival (March 1- 31, 2018).

### Data Sources

The collection and preliminary statistics of the mobile big data were carried out by China Mobile Communications Corporation (CMCC) according to a signed cooperation agreement. The mobile big data on app user, geographic location, and mobile signaling were obtained from the PaaS platform and stored and analyzed using the Hadoop computing framework in the cloud computing environment of CMCC. Preliminary aggregated statistics, such as app user numbers, the geographical distribution of app use and HTC at subdistrict scale across different time periods, and the distribution of destination cities during the Spring Festival in 2018 were exported from the PaaS platform for further statistical analysis. The male population data at the subdistrict level were obtained from the Shenzhen Bureau of Statistics.

The GIS data (GeoJSON format, Geospatial Standard GS {2024} 0650) for the map of China were downloaded from the National Platform for Common Geospatial Information Services. Areas with extremely sparse populations, such as Guangming Farmland and Inner Lingding Island, were excluded from this study and were not displayed on the Map of Shenzhen, as they have minimal impact on the overall distribution and population mobility patterns.

### Study Size

Given that mobile big data can potentially obtain information of all target app users, this study encompassed the entire target population, assuming all gay dating app users are generally men who have sex with men. CMCC counted all users of the 3 target apps (Blued, Jack'd, or Zank) in Shenzhen using unique phone numbers, allowing effective deduplication. Although dual SIM cards enable 2 phone numbers to be used on a single phone, such instances are relatively rare and unlikely to significantly impact the results given the large population size.

### Data Analysis

#### Population Estimates

The total population of men who have sex with men was estimated by combining the percentage (P1) of the target 3 apps among all apps of this kind based on surveillance data, the market share (P2) of CMCC, and the known number of the men who have sex with men users (S) of the target 3 apps (Blued, Jack'd, or Zank) from the survey. The total population of men who have sex with men (N) was then estimated using the formula.



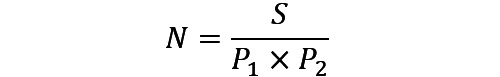



where *S* is the number of users of Blued, Jack'd, and Zank in this survey; *P*_1_ is the percentage of users of Blued, Jack'd, and Zank among all similar apps based on surveillance data; and *P*_2_ is the market share of CMCC, representing the proportion of users in Shenzhen who are served by this telecom provider.

#### Spatial Analysis

Based on the acquired geographic location data at the subdistrict scale, ArcGIS (version 10.7; Esri), a professional mapping software for spatial geographic information systems, was used to create choropleth thematic maps of the spatial distribution of the population of men who have sex with men during different time periods. Spatiotemporal autocorrelation analysis was conducted, along with the identification of spatial hot spots in the distribution of app-using men who have sex with men across these time periods. In addition, the distribution of their visits to CDCs and the 258-RWG during different time periods was compared with the distribution of app-using at the subdistrict level.

As one of the most widely used spatial autocorrelation measures, Global Moran I was used to identify spatial autocorrelation. The Global Moran I index was used to assess the spatial autocorrelation of the app use and HTC of the app-using men who have sex with men, with value ranges from –1 to 1, where approximately 0-1 indicates clustering; 0=randomness; and approximately –1 to 0=dispersion. Meanwhile, the High/Low Clustering (Getis-Ord General G) tool was applied to measure the clustering of high or low values of the app using population. The Getis Ord Gi statistic, an index of local spatial autocorrelation, was also used to identify statistically significant spatial clusters of high values (hot spots) and low values (cold spots).

In addition, we used Geodetector software (Chinese Academy of Sciences) to measure and attribute the spatially stratified heterogeneity (SSH) feature of gay dating app use in Shenzhen, characterizing the differences both within and between geographic strata. Geodetector uses the *q*-statistic index to quantify SSH and to assess the interaction outcomes between one or more independent factors. A *q* value of 0 represents an absence of SSH, while a *q* value of 1 represents perfect SSH between strata.

#### Statistical Analysis

Pearson chi-square test and Kruskal-Wallis rank sum test were used to assess the differences in age distribution and median age among users of the 3 apps. The post hoc Dunn test was further applied to perform pairwise comparisons across the 3 apps. All statistical analyses were performed using R software (version 4.4.1; R Foundation for Statistical Computing).

### Ethical Considerations

Given that only aggregated data was exported from the PaaS platform for further spatial and statistical analysis, no private information was collected, and no informed consent was required. Privacy protection was fully guaranteed. In addition, this research was reviewed and approved by the Medical Ethics Committee of the Shenzhen Center for Disease Control and Prevention (review number R2017010).

### Result

#### Population of App-Using Men Who Have Sex With Men

From September 2017 to August 2018, a total of 158,387 people used one of the 3 apps: Blued, Jack'd, and Zank, with the majority using Blued (n=117,595, 74.25%) and Jack'd (n=39,040, 24.65%; Table 1). According to our surveillance data, 98.2% of men who have sex with men who reported using a GSN app used one of the 3 target apps, suggesting the population of users collected from these three apps is relatively representative of the entire app-using population of men who have sex with men. Considering that China Mobile Limited held a 60% share of the personal mobile market in 2018 [33], the population of app-using men who have sex with men in Shenzhen was estimated to be 268,817 during 2017-2018, using the formula outlined in the Methods section.

**Table 1:** Population of app users in Shenzhen from September 2017 to August 2018 (N=158,387).

Gay dating app use	Users, n (%)
Blued	117,595 (74.25)
Jack'd	39,040 (24.65)
Zank	79 (0.05)
Blued + Jack'd	1468 (0.93)
Blued + Zank	197 (0.12)
Jack'd + Zank	1 (0.00)
Blued + Jack'd + Zank	7 (0.004)

#### Characteristics of App Use

Of the 67,050 app-using men who have sex with men with age information available, the median age was 30 (IQR 25-38) years (Table 2). There was a significant difference in the median age of users across the 3 apps (*χ*^2^_2_=4.97; *P*<.001). The post hoc Dunn test revealed a significant difference in median age between Blued and Jackd (*P*<.001), but no significant differences were observed between Blued and Zank (*P*=.41), Zank and Jackd (*P*=.07). Additionally, the age distribution among users of the 3 apps was significantly different (*χ*^2^_6_=534.4; *P*<.001). Among the male population aged 15-69 years, the overall app user-to-male ratio was approximately 2.58%, with the highest ratio (3.31%) observed among those aged 25-34 years, and the lowest ratio (1.55%) observed among older adults aged 50-69 years.

**Figure 1:**
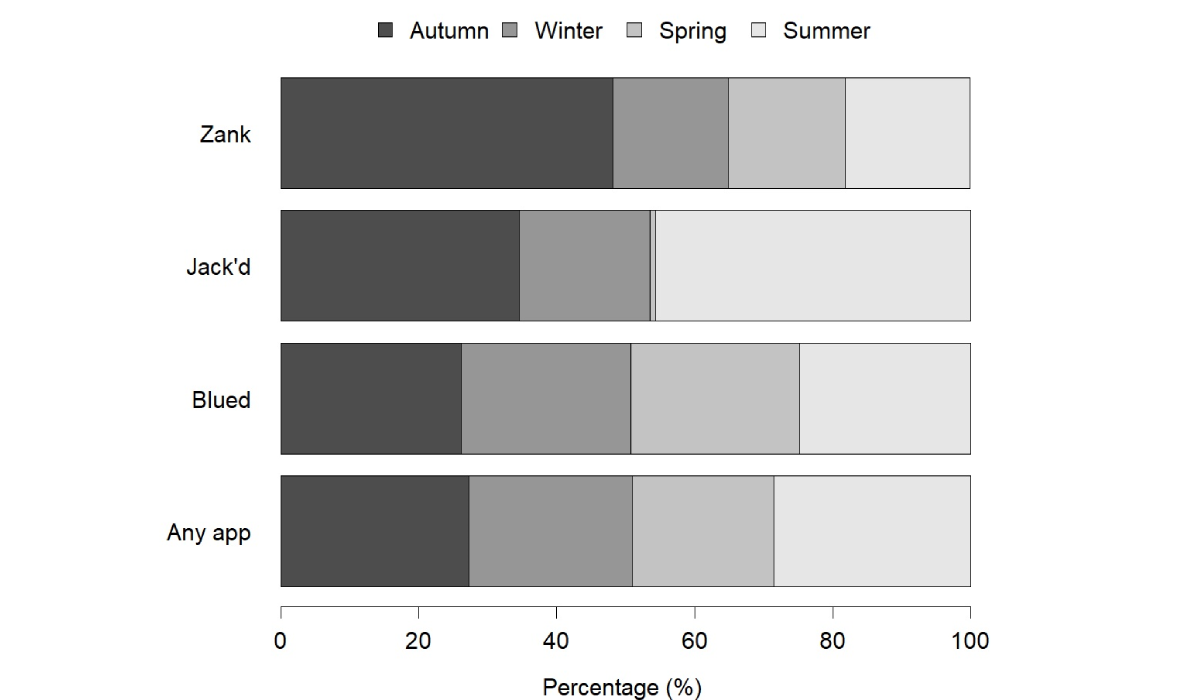
Seasonal distribution of app-using men who have sex with men in Shenzhen from September 2017 to August 2018.

The seasonal distribution of app use among men who have sex with men in Shenzhen within the study period (Figure 1) showed a slightly higher percentage of app use during summer and autumn compared to other seasons. For the specific app use, no significant seasonal trend was observed for Blued. However, a seasonal trend was observed for Jack'd and Zank. Specifically, Jack'd use was higher in the summer than in other seasons, whereas Zank use peaked in the autumn relative to other seasons.

**Table 2:** Age distribution of app-using men who have sex with men in Shenzhen from September 2017 to August 2018.

Age	Blued	Jack’d	Zank	Any app	Estimated users	Male population, n (%)	App user (male), %
All	46,969	20,738	181	67,050	158,387	6,129,495	2.58
15-24 years, n (%)	8971 (19.10)	5228 (25.21)	19 (10.50)	14088 (21.01)	33,279 (21.01)	1,715,435 (27.99)	1.94
25-34 years, n (%)	22,388 (47.67)	8180 (39.44)	92 (50.83)	30,191 (45.03)	71,318 (45.03)	2,157,632 (35.20)	3.31
35-49 years, n (%)	13,586 (28.93)	6563 (31.65)	65 (35.91)	19,998 (29.83)	47,240 (29.83)	1,833,102 (29.91)	2.58
50-69 years, n (%)	2024 (4.31)	767 (3.70)	5 (2.76)	2773 (4.14)	6550 (4.14)	423,326 (6.91)	1.55
Median (IQR)	30 (26-37)	30 (24-38)	31 (27-38)	30 (25-38)	—^a^	—	—

^a^Data not available.

#### Distribution and Hot Spots of App Use

**Figure 2:**
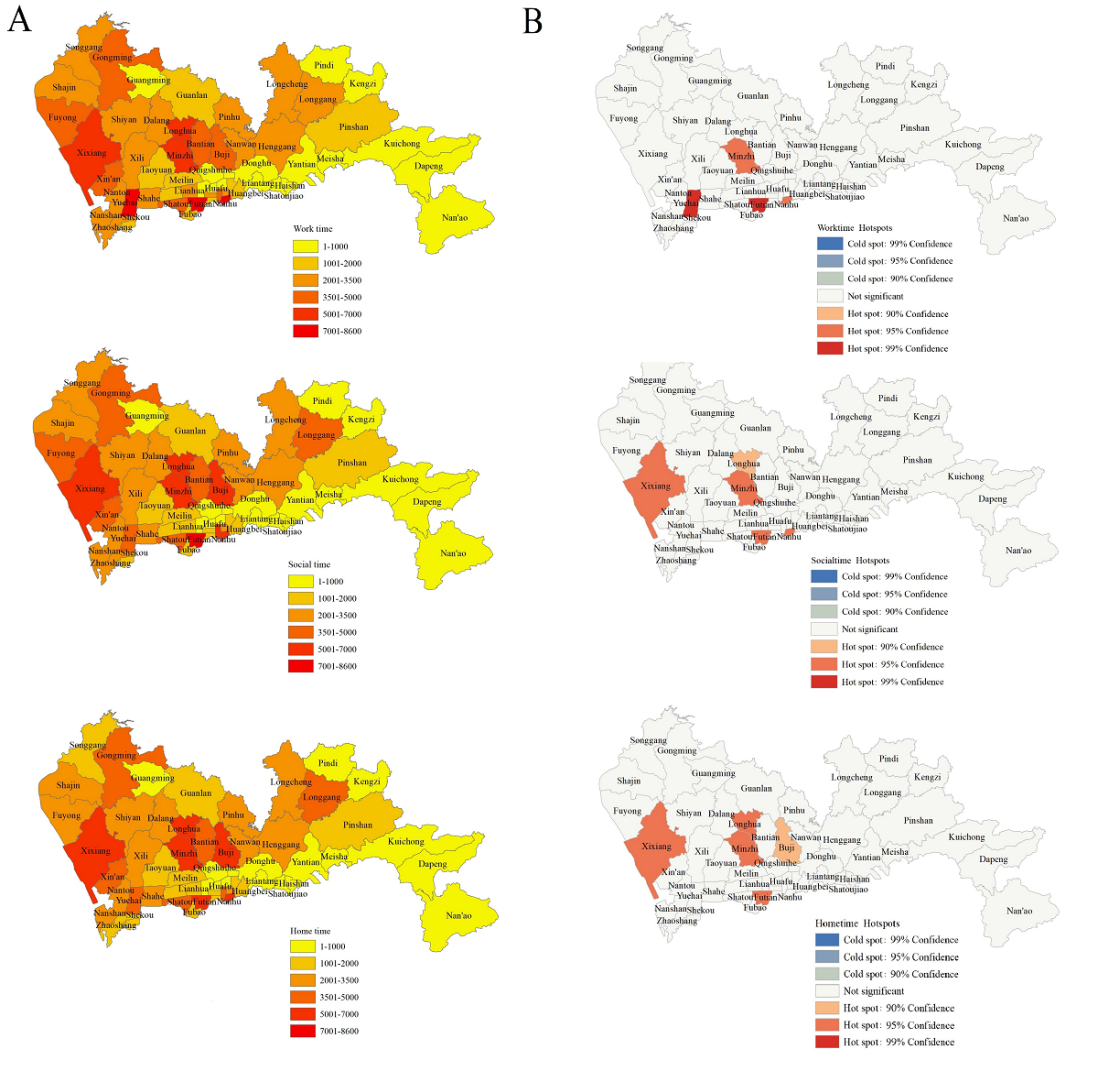
Geographic distribution and hot spots of app-using prevalence by time periods.

The geographic distribution of the gay dating app–using population during the 3 time periods was mapped at a subdistrict scale (Figure 2A). The population was relatively greater during work time (n=152,972) and social time (n=152,521) compared to home time (n=142,719). For work time, the largest population of app use was observed in the Futian, Yuehai, and Nanhu subdistricts (more than 7000), followed by Minzhi, Longhua, and Xixiang (n=5000-7000). During the social time, the highest population was observed again in the Futian subdistrict (more than 7000), followed by Minzhi, Nanhu, Xixiang, Longhua, and Buji subdistricts (n=5000-7000). For home time, the highest population was located in Xixiang, Minzhi, Futian, Longhua, Buji, and Nanhu subdistricts (n=5000-7000).

The Moran indexes derived from spatial autocorrelation analysis were all above 0 and statistically significant for the 3 time periods, suggesting the geographic distribution of app-using men who have sex with men in Shenzhen exhibited a clustered pattern (Table 3). Furthermore, the High/Low Clustering (Getis-Ord General G) statistics demonstrated significant high-high clustering for all 3 time periods (before: *z* score=3.426; *P*=.001; during *z* score=3.072; *P=*.002*;* after: *z* score=2.717; *P*=.007), indicating areas with high values of app-using population were spatially clustered.

Hot spot analysis identified Yuehai and Futian as the primary hot spots for app-using populations during work time, each with a Gi_Bin Score of 3 (*P*=.004 and *P*=.002). Meanwhile, Nanhu and Minzhi were also recognized as hot spots with a Gi_Bin Score of 2 (*P*=.01 and *P*=.04; Figure 2B; Multimedia Appendix 1). During social time, Futian, Minzhi, Nanhu, and Xixiang were identified as hot spots, each with a Gi_Bin Score of 2 (*P*=.01, *P*=.02, *P*=.02, and *P*=.02). Subsequently, for home time, Xixiang, Minzhi, Futian, and Longhua were recognized as hot spots, each with a Gi_Bin Score of 2 (*P*=.01, *P*=.02, *P*=.02, and *P*=.03) for home time.

**Table 3:** Moran indexes of app use by time periods in Shenzhen (from September 2017 to August 2018).

Statistics	Home time (2 AM-6 AM)	Social time (8 PM-2 AM)	Work time (9 AM-6 PM)
Moran index	0.272	0.238	0.190
*z* score	3.210	2.837	2.338
*P* value	.001	.005	.02
Observed General G	0.022	0.021	0.021
Expected General G	0.018	0.018	0.018
*z* score	3.426	3.072	2.717
*P* value	.001	.002	.007

#### Distribution and Hot Spots of HTC

**Figure 3:**
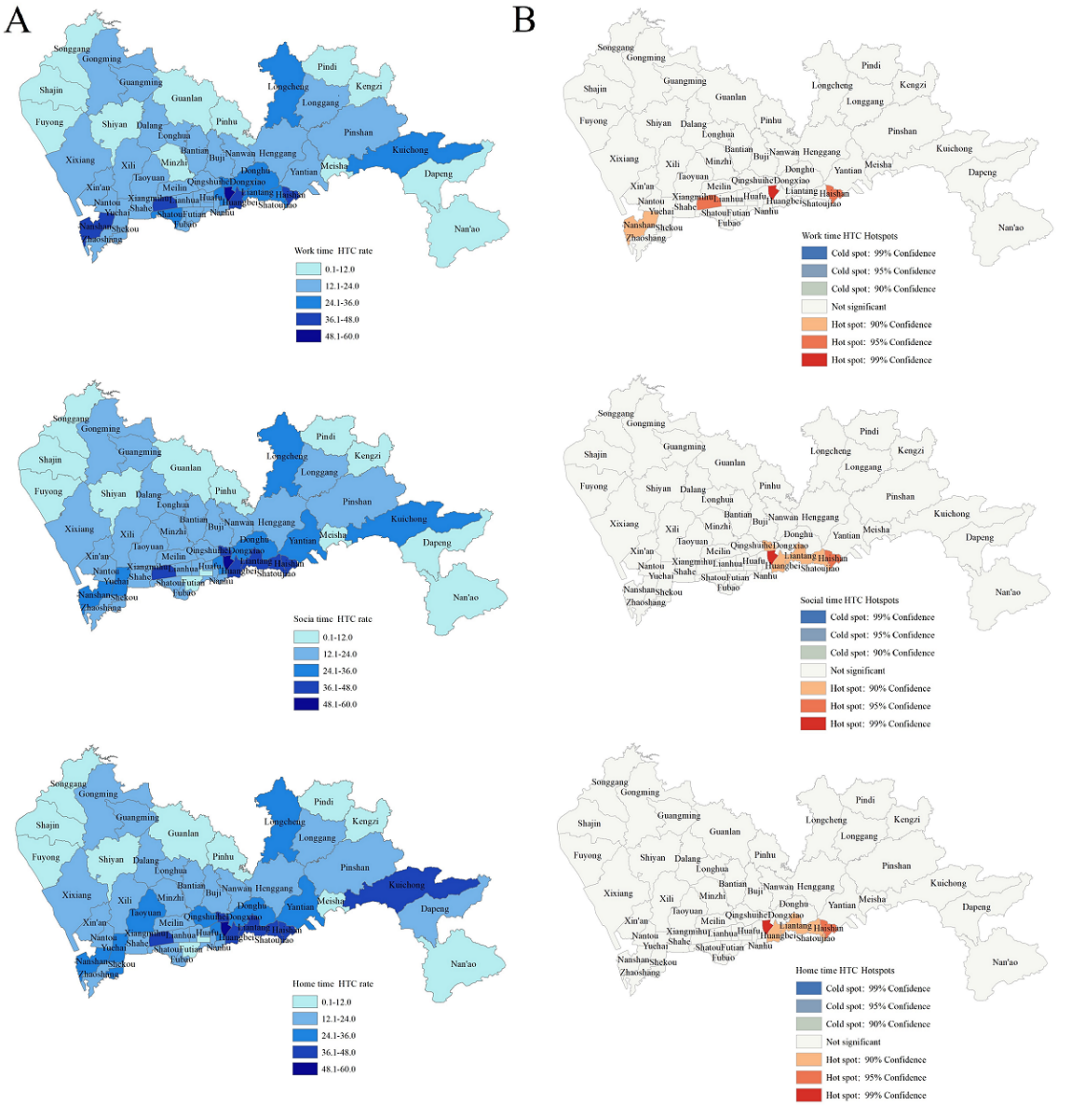
Geographic distribution and hot spots of HTC prevalence by time periods. HTC: HIV testing and counseling.

The overall prevalence of HTC among app-using men who have sex with men aged 15-69 years in Shenzhen was 17.1%, with higher rates observed during home time (18.9%) compared to social time (17.8%) and work time (17.7%). The geographic distribution of HTC prevalence was uneven at the subdistrict scale in Shenzhen (Figure 3A), with higher prevalence reported in Cuizhu, Xiangmihu, Haishan, Nanshan, and Huangbei subdistricts (>36%) during work time. For social time, a higher prevalence was observed in Cuizhu subdistrict (>48%), followed by Haishan, Shatoujiao, Liantang, Dongxiao, Huangbei, and Xiangmihu subdistricts (>36%). A similar distribution was noted for home time with the addition of Kuicheng subdistrict (>36%). The Moran indexes were statistically significant across all 3 time periods (Table 4). The High/Low Clustering (Getis-Ord General G) statistics indicated the distribution of high values of HTC prevalence was spatially clustered for both home time and social time, but not significant for work time. Hot spot analysis of the HTC prevalence revealed most of the hot spots were concentrated in the Cuizhu subdistrict with a Gi_Bin Score of 3 (*P*=.001), as well as in Xiangmihu and Haishan subdistricts, with Gi_Bin Score of 2 (*P*=.02 and *P*=.03) during the work time (Figure 3B; Multimedia Appendix 2). Similarly, the hot spots of the HTC prevalence during social time and home time were predominantly clustered in the Cuizhu subdistrict with a Gi_Bin Score of 3 (*P*=.002 and *P*=.002), and in the Haishan subdistrict with a Gi_Bin Score of 2 (*P*=.04 and *P*=.04; Figure 3B).

**Table 4:** Moran indexes of HTCa prevalence among app-using men who have sex with men in Shenzhen (from September 2017 to August 2018).

Statistics	Home time (2 AM-6 AM)	Social time (8 PM-2 AM)	Work time (9 AM-6 PM)
Moran index	0.456	0.450	0.345
*z* score	5.255	5.200	4.060
*P* value	<.001	<.001	<.001
Observed General G	0.020	0.020	0.019
Expected General G	0.018	0.018	0.018
*z* score	3.106	3.204	2.370
*P* value	.002	.001	.02

^a^HTC: HIV testing and counseling.

#### SSH of App Use and HTC

The Geodetector index of *q* statistics was also used to measure the stratified heterogeneity of app use and HTC across different strata (time period, district, subdistrict, and the proportion of non-Hukou population; Table 5). For both the prevalence of app use and HTC, the *q* statistics and *P* values indicated significant heterogeneity in administrative districts (*P*=.006 and *P*<.001), subdistricts (*P*<.001 and *P*<.001), and the percentage of non-Hukou migrants (*P*<.001 and *P*<.001). However, no significant heterogeneity was observed across different time periods. Luohu district demonstrated the highest risk for both app use and HTC. Nanhu subdistrict, along with subdistricts with 50%-60% non-Hukou migrants, exhibited a higher risk for app use than other areas. Meanwhile, the Cuizhu subdistrict and those subdistricts with 40%-50% non-Hukou migrants reported the highest rates for HTC.

**Table 5:** Stratified heterogeneity of app use and HTCa in Shenzhen (from September 2017 to August 2018).

Factors			*q* statistic		*P* value		Highest risk detector
**App use**							
	Time period	0.004		.71		—^b^	
	Subdistrict	0.949		<.001		Nanhu	
	District	0.147		.006		Luohu	
	Non-Hukou migrants (%)	0.148		<.001		L6 (50%-60%)	
**HTC**							
	Time period	0.002		.80		—	
	Subdistrict	0.958		<.001		Cuizhu	
	District	0.420		<.001		Luohu	
	Non-Hukou migrants (%)	0.378		<.001		L5 (40%-50%)	

^a^HTC: HIV testing and counseling.

^b^Data not applicable.

#### Population Mobility

**Figure 4:**
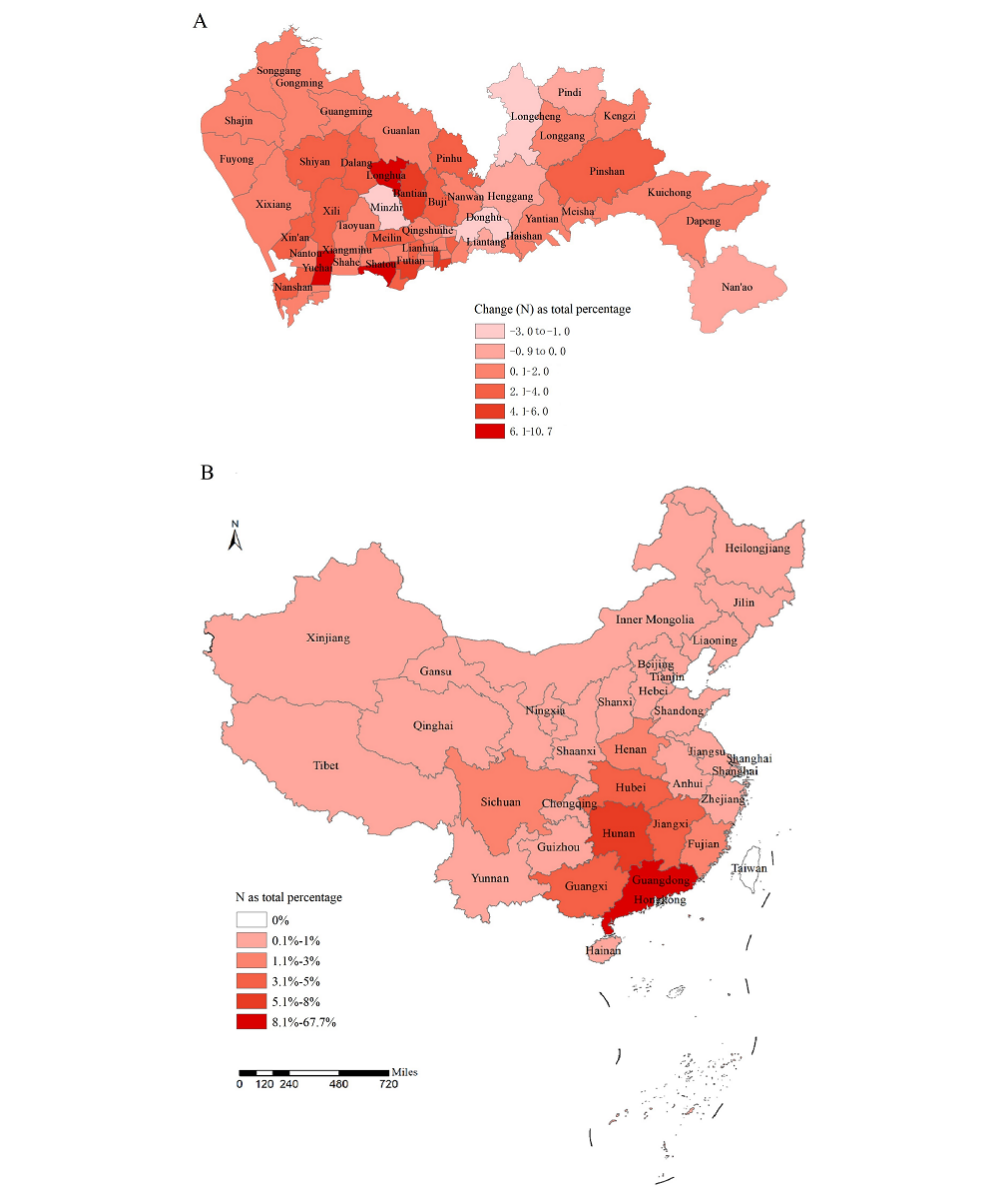
Mobility of app-using men who have sex with men in Shenzhen during the Spring Festival in 2018.

**Figure 5:**
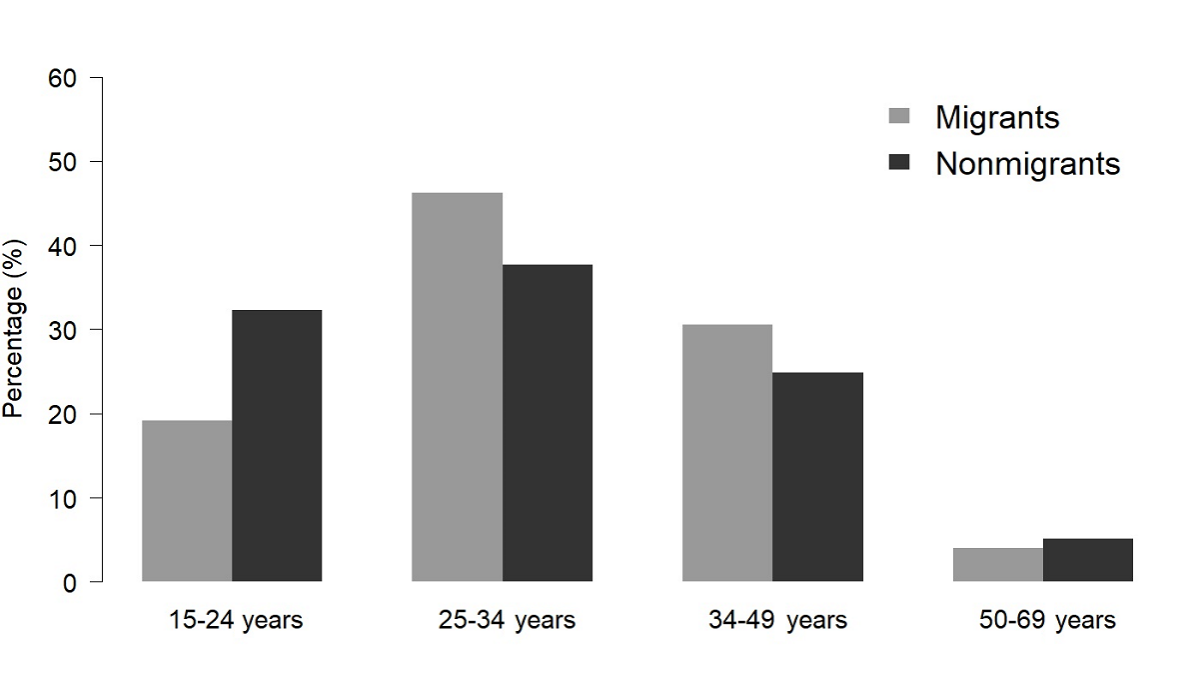
Age distribution of app-using men who have sex with men in Shenzhen during the Spring Festival in 2018.

The geographical distribution of app-using men who have sex with men in Shenzhen before, during, and after the Spring Festival of 2018 was mapped (Multimedia Appendix 3). The results showed that prior to the Spring Festival, app-using men who have sex with men were mostly located in Xixiang, Yuehai, and Longhua subdistricts. During the Spring Festival, those who stayed in Shenzhen were relatively concentrated in Xixiang, Longhua, and Minzhi subdistricts. After the Spring Festival, app-using men who have sex with men returned to clustered in Xixiang, Yuehai, Longhua, and Minzhi subdistricts. For the population change of app-using men who have sex with men before and after the Spring Festival, the largest inflow occurred in the Yuehai, Shatou, and Longhua subdistricts, while the most significant outflow was observed in the Longcheng and Donghu subdistricts (Figure 4A). Additionally, the population mobility of app-using men who have sex with men during the Spring Festival was mapped based on changes in geographical location at the subdistrict scale (Figure 4B). During the Spring Festival, 60,202 (38%) app-using men who have sex with men left Shenzhen, while 37,756 (62.7%) of them returned after the holiday. The distribution of the destination cities for these app-using men who have sex with men demonstrated a relatively centralized flow to other cities in China, with the largest mobility directed toward other cities within Guangdong province (67.7%). There was also lower mobility to Hunan province (7.9%) and other adjacent provinces, such as Jiangxi, Guangxi, and Hubei Provinces (approximately 3%-5%). In terms of age distribution, a higher proportion of those who left Shenzhen were aged 25-34 years and 35-49 years. In contrast, those who stayed were predominantly younger men who have sex with men aged 24 years or younger, as well as older adults aged 50-69 years (*P*<.001; Figure 5).

## Discussion

### Principal Findings

This study represents the first longitudinal observational research on the GSN app use, examining its genuine population size, spatiotemporal and behavioral patterns, and mobility through mobile big data in mainland China. This study found Shenzhen has a larger population of men who have sex with men than expected. The geographic distribution of app-using men who have sex with men in Shenzhen exhibited a clustered pattern in central and western Shenzhen, while their HTC uptake was more concentrated in central-eastern Shenzhen. Moreover, both relatively high internal and interprovincial mobility was observed among those app-using men who have sex with men. The findings provided a comprehensive view of the behaviors and dynamics of app-using men who have sex with men, offering valuable insights to inform enhanced prevention and intervention strategies and guide resource allocation. Moreover, our research demonstrates the potential of mobile big data to address critical gaps often overlooked by traditional research methods, providing more accurate, timely, and comprehensive insights into public health and disease prevention.

### App Use

This study implied a large population of app-using men who have sex with men in Shenzhen from September 2017 to August 2018, surpassing previous estimates [31]. Moreover, the annual estimate of app-user or male ratio (2.58% among males aged 15-69 years) was relatively high in Shenzhen, compared to the daily estimates of Beijing and Shanghai (0.82% and 0.56% among males aged 18-64 years) from a concurrent experimental study [28]. Literature implied that overestimation might exist in economically developed countries characterized by higher urbanization, higher education levels, more in-migrants, and larger working-age populations [34]. Nonetheless, the population of men who have sex with men may have continued to grow along with the growth of the general population in Shenzhen since 2018, underscoring the increasing need for HIV prevention and intervention efforts targeting men who have sex with men in the region.

Our previous surveillance data indicated a rapid increase in app use among men who have sex with men in Shenzhen since 2016, with Blued being the most frequently used app, consistent with findings from previous data in Shenzhen and other cities [12,26]. An internet survey suggested men who have sex with men in southern China used Jack'd more frequently [35]. Founded in 2010, Jack'd has experienced significant growth, particularly among young men who have sex with men, boasting over 5 million users worldwide since 2014 [36]. Notably, nearly half of the app users were aged between 25-34 years, which aligns with other internet surveys [8,14,35]. In Shenzhen, the median age of new infections was 31 years in 2011-2019, with cases younger than 30 years more frequently observed within molecular transmission clusters [37]. Thus, tailored strategies are needed to enhance the effectiveness of HIV prevention initiatives targeting these younger men who have sex with men.

### Spatiotemporal Distribution and SSH

The temporal distribution pattern of app-using men who have sex with men implied that these men who have sex with men tend to use the apps more frequently during work time (daytime) and social time compared to home time. It is reasonable to assume that app users initially seek potential partners through the internet before transitioning to offline social networking if they find a desirable match from the app. During home time, however, it is likely that most users prefer to rest. This finding suggested a clear temporal distinction: a more active search for connections during work and social hours, and a more relaxed, less frequent engagement during home time. Regarding the geographical distribution, app-using men who have sex with men in Shenzhen exhibited spatial clustering in several subdistricts (Futian, Yuehai, Nanhu, Xixiang, Minzhi, and Longhua) at different time periods. Most clusterings occurred in Nanshan District for work time, Futian District for social time, and Baoan District for home time. This study, for the first time, revealed the spatial heterogeneity for gay dating app–using men who have sex with men in Shenzhen, suggesting that these men who have sex with men were unevenly distributed both at the subdistricts and district levels, as well as among the Hukou residents. As app-using men who have sex with men were more likely to have multiple sexual partners, practice receptive roles in anal sex, and use recreational drugs [12]. By recognizing the different contexts in which men who have sex with men engage with dating apps, public health initiatives can be designed to effectively reach this population at the right times and in the right settings. Traditional HIV prevention services (eg, condoms and lubricants distribution) and interventions (eg, antidrug campaigns) should be delivered more precisely to the identified hot spots based on both temporal and geographical distribution.

Meanwhile, compared to the distribution of app-using men who have sex with men, the prevalence of HTC among this population in Shenzhen showed obvious inconsistencies. HTC rates were concentrated in different subdistricts (eg, Cuizhu, Haishan, Xiangmihu, Nanshan, Shatoujiao, and Huangbei), with the highest clustering observed in Luohu, Futian, and Yantian Districts during social and home time. For work time, HTC is also clustered in Nanshan District. These findings implied that men who have sex with men might prefer to seek HTC services in central districts of Shenzhen, which had higher accessibility to health care [38], or choose these areas for convenience after engaging in high-risk sexual behavior during offline dating or gatherings.

Given the disparities in the distribution of app use and HTC service, it is crucial to allocate more HIV-related health care services (eg, HIV testing and counseling) to western Shenzhen, particularly in subdistricts of Baoan District. Furthermore, the mismatched distribution of app use and HTC and the availability of fee HTC service highlighted the need for outreach initiatives that extend beyond traditional health care settings. Implementing mobile clinics or pop-up testing stations in hot spots or underserved areas could help bridge the HIV testing gap.

Although our previous study implied that app-using men who have sex with men were more likely to take HIV tests, the prevalence of lifetime and recent HIV tests in the past year (70.2% and 50.2%) remained significantly below the UNAIDS target [39]. Furthermore, it is reported that the majority (40%) of the app-using men who have sex with men preferred to take HIV tests at CDCs and community-based organizations (eg, 258-RWG) due to stigma and confidential concerns [39]. Besides, traditional offline HTC services are also available at gay social venues, voluntary counseling and testing centers, or clinics, while the uptake of these services has not been optimal. Therefore, it is essential to prioritize location-tailored strategies for expanding HIV testing, particularly in identified geographical hot spots. Concurrently, innovative web-based prevention initiatives targeting app-using men who have sex with men emerged as a promising alternative. For instance, Blued launched an HIV testing campaign in 2015, which recommended the nearest health care facility or testing service for app users [27]. In light of these insights, future strategies could leverage GSN apps and smartphone tools to promote HTC services, ultimately contributing to the achievement of the first UNAIDS “95” goal.

### Migration During the Spring Festival

During the 2018 Spring Festival, more than one-third of app-using men who have sex with men left Shenzhen, highlighting the high mobility of this population. A prior study also reported that about one-fourth of the gay dating app-using men who have sex with men lived in Shenzhen for less than 1 year [12]. The distribution of the destination cities for app-using men who have sex with men demonstrated a relatively centralized flow, with greater internal movement within Guangdong province and less interprovincial mobility to neighboring provinces such as Hunan, Jiangxi, Guangxi, and Hubei. Research suggested that immigrants from Jiangxi, Shaanxi, and Hubei provinces were the main sources of the predominant CRF01_AE subtype in Shenzhen [18]. Local statistics also reported that the immigrants in Shenzhen mainly came from Guangdong (35%), followed by Hunan, Sichuan, Guangxi, Hubei, and Jiangxi. A nationwide study further supported these findings, identifying Hunan, Guangxi, Hubei, and Jiangxi Provinces as the main source provinces for migrant men who have sex with men in Guangdong [28]. During the Spring Festival, the most significant changes in the population of men who have sex with men were observed in the Yuehai, Shatou, and Longhua subdistricts. Therefore, it is crucial to strengthen targeted and innovative prevention and intervention efforts in these areas to address the unique challenges faced by migrant men who have sex with men, such as app-based notifications or location-based services, to provide timely and targeted HIV prevention messages and services, particularly during periods of high mobility.

### Limitations

First, the application of mobile internet has developed rapidly in China, especially in the last 5 years, which may have a significant impact on the behavioral characteristics of men who have sex with men, it would be valuable to collect more recent data to examine the evolution of the trend in the future. Second, as the study is based on mobile big data, there may be inherent biases in estimating the true population size. Third, regarding the geographical difference in app use and HTC, limited demographic data prevented us from differentiating the impact of resource availability from user selection bias. Moreover, it is difficult to distinguish male sex workers and noncommercial men who have sex with men, who may exhibit different HIV-related behaviors [40]. Therefore, future studies with more demographic factors (eg, occupation and income) and other socioeconomic determinants, are needed to differentiate selection bias. Finally, although gay dating app users are generally assumed to be men who have sex with men, it is possible that a small number of non–men who have sex with men individuals may also use the app for research purposes or by chance. However, as this group constitutes a negligible portion of the overall population, their presence is unlikely to affect the results significantly and can therefore be disregarded.

### Conclusions

Shenzhen has a large population of men who have sex with men using gay dating apps, predominantly aged 15-34 years. The uneven spatiotemporal distribution of app-using men who have sex with men and their HIV testing or counseling, as well as the mismatch between them, emphasizes the need to adapt the traditional venue-based prevention and intervention strategies to the identified hot spots area and the need for outreach initiatives that extend beyond traditional health care settings. Given the relatively high internal and interprovincial mobility of this population, leveraging smartphone-based behavioral monitoring could provide valuable insights for enhanced and innovative prevention and intervention strategies.

## Appendix

Multimedia Appendix 1 Hot spots of app-using population by time periods in Shenzhen (September 2017-August 2018).

Multimedia Appendix 2 Hot spots of HTC prevalence by time periods in Shenzhen (September 2017-August 2018).

Multimedia Appendix 3 Geographical distribution of app-using MSM in Shenzhen (A) before, (B) during, and (C) after the Spring Festival of 2018.

## Abbreviations

258-RWG: 258-Rainbow Working Group
CDC: Center for Disease Control and Prevention
CMCC: China Mobile Communications Corporation
GSN: geosocial networking
HTC: HIV testing and counseling
PaaS: platform as a service
PrEP: pre-exposure prophylaxis
SSH: spatially stratified heterogeneity

## Data Availability

The datasets used during this study are available from the corresponding author on reasonable request.
