# Supplementary material for: Spatiotemporal and Behavioral Patterns of Men Who Have Sex With Men Using Geosocial Networking Apps in Shenzhen From Mobile Big Data Perspective: Longitudinal Observational Study
Source: J Med Internet Res. 2025 Mar 20;27:e69569. doi: 10.2196/69569 (PMC11969128; doi:10.2196/69569)
Supplement: Multimedia Appendix 1 [file jmir_v27i1e69569_app1.docx]

**Table S1.** Hot spots of app-using population by time periods in Shenzhen (September 2017-August 2018).

| **Time period** | **Subdistrict** | **Gi Z-Score** | **Gi P Value** | **Gi_Bin** |
| --- | --- | --- | --- | --- |
| Work time | Yuehai | 2.852 | 0.004 | 3 |
|  | Futian | 3.095 | 0.002 | 3 |
|  | Nanhu | 2.520 | 0.012 | 2 |
|  | Minzhi | 2.112 | 0.035 | 2 |
| Social time | Futian | 2.540 | 0.011 | 2 |
|  | Minzhi | 2.387 | 0.017 | 2 |
|  | Nanhu | 2.345 | 0.019 | 2 |
|  | Xixiang | 2.308 | 0.021 | 2 |
| Home time | Xixiang | 2.542 | 0.011 | 2 |
|  | Minzhi | 2.322 | 0.020 | 2 |
|  | Futian | 2.274 | 0.023 | 2 |
|  | Longhua | 2.172 | 0.030 | 2 |
