# Supplementary material for: Spatiotemporal and Behavioral Patterns of Men Who Have Sex With Men Using Geosocial Networking Apps in Shenzhen From Mobile Big Data Perspective: Longitudinal Observational Study
Source: J Med Internet Res. 2025 Mar 20;27:e69569. doi: 10.2196/69569 (PMC11969128; doi:10.2196/69569)
Supplement: Multimedia Appendix 2 [file jmir_v27i1e69569_app2.docx]

**Table S2.** Hot spots of HTC prevalence by time periods in Shenzhen (September 2017-August 2018).

| **Time period** | **Subdistrict** | **Gi Z-Score** | **Gi P Value** | **Gi_Bin** |
| --- | --- | --- | --- | --- |
| Work time | Cuizhu | 3.280 | 0.001 | 3 |
|  | Xiangmihu | 2.408 | 0.016 | 2 |
|  | Haishan | 2.177 | 0.029 | 2 |
| Social time | Cuizhu | 3.093 | 0.002 | 3 |
|  | Haishan | 2.047 | 0.041 | 2 |
| Home time | Cuizhu | 3.024 | 0.002 | 3 |
|  | Haishan | 2.021 | 0.043 | 2 |
