# Supplementary figures and images for: Spatiotemporal and Behavioral Patterns of Men Who Have Sex With Men Using Geosocial Networking Apps in Shenzhen From Mobile Big Data Perspective: Longitudinal Observational Study
Source: J Med Internet Res. 2025 Mar 20;27:e69569. doi: 10.2196/69569 (PMC11969128; doi:10.2196/69569)

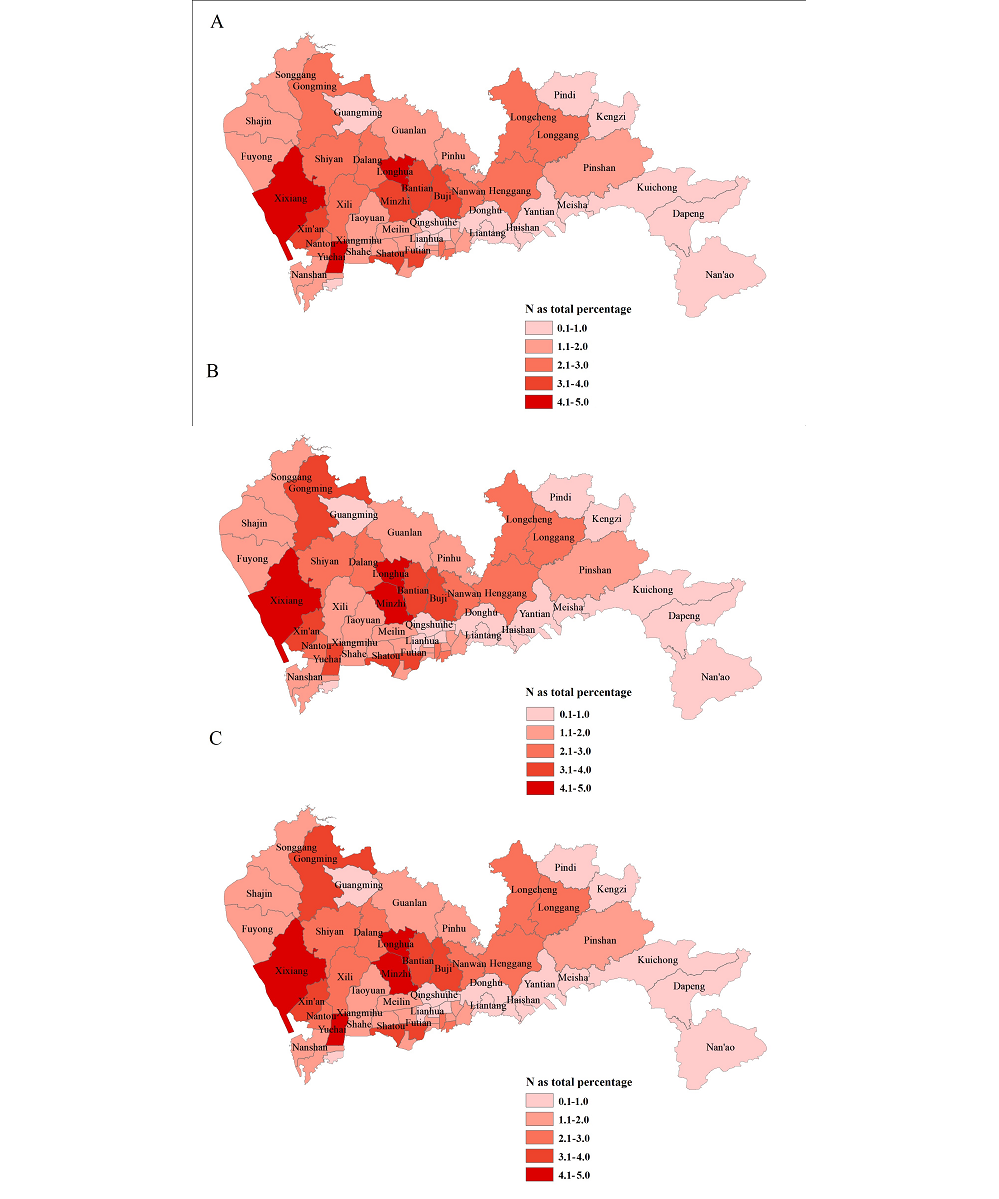

Supplement: Multimedia Appendix 3 [file jmir_v27i1e69569_app3.png]
